# Supplementary material for: Eucommia ulmoides Flavones as Potential Alternatives to Antibiotic Growth Promoters in a Low-Protein Diet Improve Growth Performance and Intestinal Health in Weaning Piglets
Source: Animals (Basel). 2020 Oct 30;10(11):1998. doi: 10.3390/ani10111998 (PMC7694009; doi:10.3390/ani10111998)
Supplement: Supplementary file 1 [file animals-10-01998-s001.pdf]

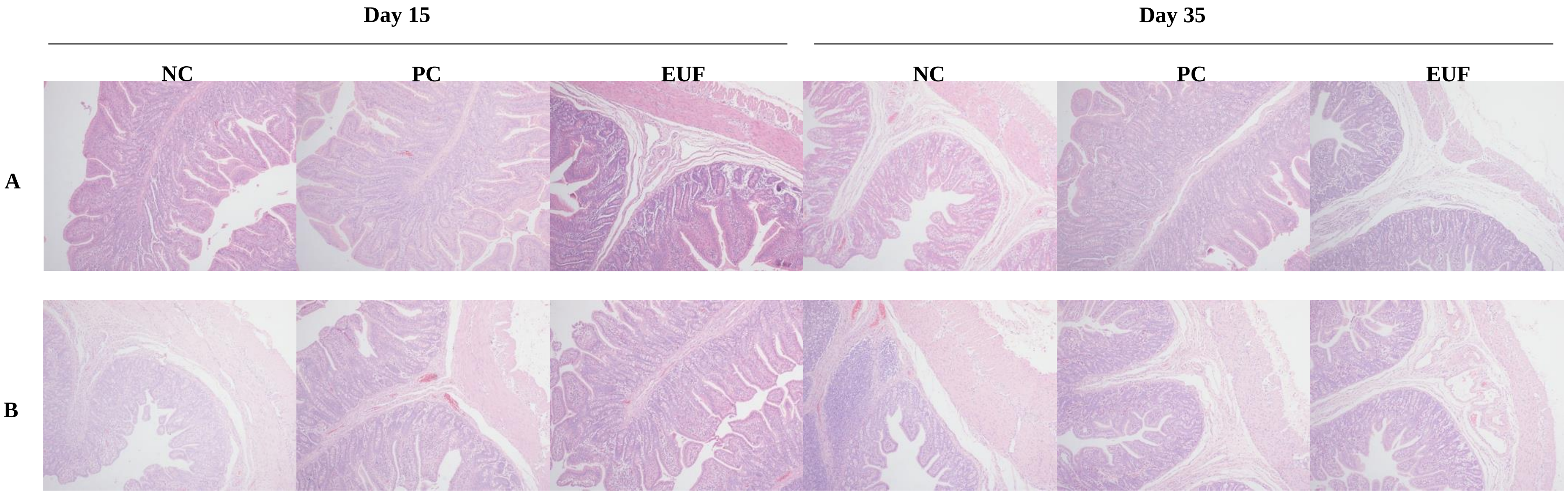

Figure S1. The representative images of scanning electron microscopy in the jejunum (A) and ileum (B) of piglets (magnification × 40). NC = negative control, low-protein basal diet no antibiotics included; PC = positive control, low-protein Basal diet + antibiotics (75 mg/kg quinocetone, 20 mg/kg virginomycin and 50 mg/kg aureomycin); EUF = *Eucommia ulmoides* flavones, low-protein Basal diet + 0.01% EUF. Values are the mean ± SEM, n = 8 per treatment group.
